# Evaluation of Bioactive Compounds and Bioactivities in Plum (*Prunus salicina* Lindl.) Wine

**DOI:** 10.3389/fnut.2021.766415

**Published:** 2021-11-01

**Authors:** Guoming Liu, Ping Wei, Yayuan Tang, Yiyang Pang, Jian Sun, Jiemin Li, Chuanyan Rao, Cuiqiong Wu, Xuemei He, Li Li, Dongning Ling, Xi Chen

**Affiliations:** ^1^Agro-Food Science and Technology Research Institute, Guangxi Academy of Agricultural Sciences, Nanning, China; ^2^Guangxi Key Laboratory of Fruits and Vegetables Storage-Processing Technology, Nanning, China

**Keywords:** Niuxin plum wine, fermentation condition, volatile aromas, polysaccharides, antioxidant activity, hypoglycemic activity

## Abstract

With the increase in demand of fruit wine year by year, it is necessary to develop novel fruit wine with high functional activities. *Prunus salicina* Lindl. (named as Niuxin plum) is a remarkable material for brewing fruit wine owing to its suitable sugar-acid ratio, characteristic aroma and bioactive compounds. This study intends to modify the fermentation technology, identify and quantify nutritional compositions and volatile profiles, as well as bioactive substances in Niuxin plum wine, as well as evaluate the antioxidant and hypoglycemic activities *in vitro* of major bioactive components from Niuxin plum wine. According to single-factor and orthogonal tests, the optimal fermentation conditions of 13.1% vol Niuxin plum wine should be *Saccharomyces cerevisiae* Lalvin EC1118 at 0.1% and a fermentation temperature of 20°C for 7 days. A total of 17 amino acids, 9 mineral elements, 4 vitamins, and 55 aromatic components were detected in plum wine. Polysaccharides from Niuxin plum wine (named as NPWPs) served as the major bioactive components. The NPWP with a molecular weight over 1,000 kDa (NPWP-10) demonstrated extraordinary DPPH free radical scavenging capacity and α-glucosidase inhibitory activity among all NPWPs having different molecular weight. Moreover, the structural characterization of NPWP-10 was also analyzed by high performance liquid chromatography (HPLC), fourier-transform infrared (FT-IR) and nuclear magnetic resonance (NMR) spectra studies. NPWP-10 was composed of mannose, rhamnose, arabinose, galactose and galacturonic acid with molar ratios of 2.570:1.775:1.045:1.037:1. NPWP-10 contained α-configuration as the main component and β-configuration as the auxiliary component. This study highlights NPWP-10 is an importantly biological polysaccharide from Niuxin plum wine, as well as provides a scientific basis for developing the plum wine industry.

## Graphical Abstract

Evaluation of bioactive compounds and bioactivities in plum (*Prunus salicina* Lindl.) wine. 
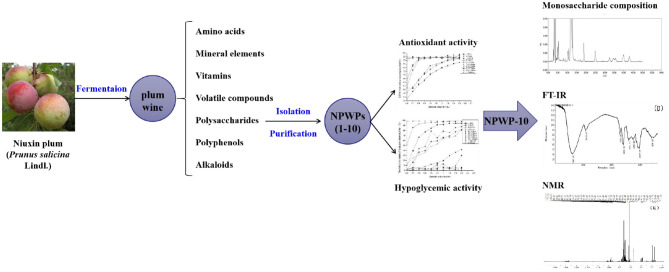

## Introduction

*Prunus salicina* Lindl., known as “Niuxin plum” in Chinese, is a crop tree species native to China, and cultivated mainly for the fresh fruit market. At present, plum also is typically processed into dried fruit, jam and jelly (1). The average weight of single Niuxin plum is about 21.57 g. Plum pulp has dietary fiber content of 1.20%, soluble sugar of 10.70%, titratable acidity of 0.65%, and soluble solid (TSS) content of 9.60% (2). Every 100 g of plum pulp contains sucrose 2.16 g, glucose 0.93 g, fructose 0.50 g, malic acid 0.81 g, quininic acid 0.16 g, citric acid 0.01 g, sorbitol 0.05 g, ascorbic acid 4.27 mg, and energy 255 kJ. A total of 63 volatile profiles, including (*E*)-2-hexenal, (*E*)-2-octenal, (*E*)-2-nonenal, decanal, 2-nonanone, 2,6,6-trimethyl-2-cyclohexene-1,4-dione, 2,4-di-tert-butylphenol, p-cymene and dipentene, were identified in plum cultivars (3, 4). The main aromatic components of Niuxin plum were esters, and the characteristic aromatic components in Niuxin plum were ethyl butyrate, ethyl caprate and isoamyl acetate. On many occasions, it has been reported that plum is a rich source of anthocyanins, flavonols, flavan-3-ols, phenolic acids (5), polysaccharides, alkaloids and chlorophyll catabolite (6). Several bioactivities of these bioactive compounds including antioxidant activity (7), anticancer (8), antidiabetic (9), and anti-inflammatory (10) were reported.

Fruit wines are defined as beverages obtained by the alcoholic fermentation of fruit juice or concentrated fruit juice, fruit pomace or concentrated fruit pomace (11). Generally, grapes are the main raw materials that have been used for wine production for the past few decades (12); however, studies have shown the suitability of tropical, subtropical and temperate fruits other than grapes like apple (13), berry (14), and plum (15) for the purpose of wine-making. In recent years, consumer demands for functional foods including fruits and their products such as fruit wine have increased substantially as they contribute to human health, nutrition and prevention of diseases. With the increase in demand of fruit wine (currently accounting for ~15–20% of global alcohol products), it is necessary to develop novel fruit wine with high functional activities. Therefore, Niuxin plum is a remarkable material for brewing fruit wine owing to its suitable sugar-acid ratio, characteristic aroma and bioactive composition. Additionally, the global annual production of plum is ~11,000,000 tons (16), estimating that nearly 10–20% of plum worldwide may be wasteful. Thus, the development of plum wine not only expands variety of food products and enhances economical value of plum, but also prevents food waste which represents an environmental problem.

To our knowledge, there have been no studies dealing with fermentation technology, bioactive components and functional activities in Niuxin plum wine, limiting its use in food industry. Accordingly, the present study intends to modify the fermentation technology of Niuxin plum wine, and to identify and quantify its nutritional ingredients such as amino acids, mineral elements and vitamins, and volatile aromas compositions as well as bioactive substances such as polyphenols, alkaloids and polysaccharides. The antioxidant and hypoglycemic activities *in vitro* of polysaccharides as major bioactive components from Niuxin plum wine were also determined. The findings of this research can offer useful references for developing diversified plum products to promote the commercial value of plum.

## Materials and Methods

### Chemicals and Reagents

*Saccharomyces cerevisiae* F33 was obtained from Tongshang International Trade Co. (Yantai, Shandong, China). *Saccharomyces cerevisiae* Lalvin71B, Lalvin EC1118, Lalvin D254, Lalvin RC212, Lalvin RC2323, Lalvin K1, Lalvin U43, Lalvin KD, and Lalvin R-HST were from Longbao Commercial and Trading Co. (Rizhao, Shandong, China). *Saccharomyces cerevisiae* Angel BV818, Angel SY, and Angel RW were provided by Angel Yeast Co. (Yichang, Hubei, China). HPLC-grade rutin, glucose, phenol, gallic acid, 4-hydroxypiperidine, acarbose, α-glucosidase (100 UN), Folin-Ciocalteu reagent, 2-diphenyl-1-picrylhydrazyl (DPPH), and p-nitrophenyl-β-d-galactopyranoside (PNPG) were provided by Sigma-Aldrich Co. (St. Louis, MO, USA). Overall, chemicals were at an analytical grade unless specified otherwise.

### Preparation of Niuxin Plum Sample

The Niuxin plum trees were planted in the hill districts of the Guangxi province of China and harvested in June 2017. For the tests, Niuxin plum fruits were rinsed and separated into pulp and seeds. The plum fruit pulp having high maturity (15.6°Brix) was stored at −20 °C for further research.

### Optimization of the Fermentation Conditions for Niuxin Plum Wine

Plum fruit pulp was pressed and extracted for juice using the LZ-1.5 juicing machine developed by Food Machinery Manufacturing Co., Jiangsu, China. The hydrolyzation of 1 L of plum juice was performed at 40°C for 2 h with 1% pectinase (Beijing Solarbio Science & Technology Co., Beijing, China). The hydrolyzed plum juice was further mixed with 178.7 g of sucrose and then sterilized under the temperature conditions of 75°C for 10 min inside the water bath. *Saccharomyces cerevisiae* was added when the juice temperature was reduced to 40°C, and fermentation was carried out at 20°C for 7 days. Three factors were introduced for single-factor testing, including *Saccharomyces cerevisiae* strain, yeast amount, and the temperature of fermentation (Table 1). Based on the results of single-factor testing, influence factors with great significance were chosen for follow-up orthogonal experiments, and the optimal conditions of fermentation for plum wine were accordingly determined. As shown in Table 2, the sensory appraisal for plum wine in the testing mentioned above was accomplished by a group staffed by ten people daily during 7 days.

**Table 1:** Single factor experiments for fermentation conditions of Niuxin plum wine.

**Factor**	**Level**	**Condition**
*Saccharomyces cerevisiae* strain	F33, Lalvin71, Lalvin EC1118, Lalvin D254, Lalvin 212, Lalvin 2323, Lalvin K1, Lalvin U43, Lalvin KD, Lalvin R-HST, Angel BV818, Angel SY, Angel RW	Fermentation temperature 20°C, yeast amount 0.10%.
Yeast amount	0.05%, 0.10%, 0.15%, 0.20%, 0.25%	Fermentation temperature 20°C, *Saccharomyces cerevisiae* Lalvin R-HST.
Fermentation temperature	16°C, 18°C, 20°C, 22°C, 24°C	*Saccharomyces cerevisiae* Lalvin R-HST, yeast amount 0.10%.

**Table 2:** Sensory evaluation standard of Niuxin plum wine.

**Sensory scores**	**Attributes of Niuxin plum wine samples**		
	**Aroma**	**Taste**	**Aftertaste**
Excellent	Obvious plum aroma and wine bouquet, all kinds of fragrance blending into harmonious whole	Sour and sweet, mellow without peculiar smell	Sweet aftertaste and last a long time
	(25–30)	(30–40)	(25–30)
Good	Plum aroma and wine bouquet, one kind of fragrance exhibiting slightly prominent	Slightly prominent sour or sweet without peculiar smell	Sweet aftertaste and last a short time
	(16–24)	(20–29)	(16–24)
Medium	Plum aroma and wine bouquet, all kinds of fra grance exhibiting disharmony	Prominent sour or sweet with a little bitterness	Bitter aftertaste and last a short time
	(10–15)	(10–19)	(10–15)
Bad	Bad smell and little plum fragrance	Too sour or sweet, even bitter	Bitter aftertaste and last a long time
	(0–9)	(0–9)	(0–9)

### Evaluation of Nutritional Compositions and Volatile Aromas in Niuxin Plum Wine

The amino acids, mineral elements, and vitamins as nutrients in Niuxin plum wine were evaluated by modified methods (17–19). The volatile compounds in Niuxin plum wine were investigated following headspace solid-phase microextraction (H-SPME) as well as gas chromatography (GC) (SCION SQ 456, Bruker Co., Madison, WI, USA) with DB-Wax capillary column (30 m × 0.25 mm × 0.25 μm, J&W Scientific, Folsom, CA, USA). Compounds with volatility properties in wine were extracted by the H-SPME method using 2-octanol as an internal standard (20). The GC conditions were as follows: EI^+^ ionization mode, 80 μA emission current, 70 eV electron energy, 250°C interface temperature, 200°C source temperature, and 1,000 V detector voltage. The temperature of the GC oven was first set to 40°C for 3 min, and it was then up-regulated by 5°C/min to 90°C and by 10°C/min to 230°C. The heating under the temperature condition of 230°C lasted for 7 min. Helium at a flow rate of 0.8 mL/min served as the carrier gas.

### Qualitative and Quantitative Analyses of Major Bioactive Components (Polysaccharides) in Niuxin Plum Wine

#### Determination of Polysaccharides Content

The content of total polysaccharides was measured using the phenol-sulfuric acid approach, in which glucose served as the extrinsic criterion (21). Results were expressed as milligram of glucose equivalents (GE)/mL wine sample (mg of GE/mL wine sample).

#### Isolation of Polysaccharides

Niuxin plum wine was first decompressed for distillation treatment under the temperature condition of 60°C and then condensed to a viscous fluid. The concentrated sample (100 mL) was mixed with 900 mL of absolute ethanol and stirred adequately, and preserved under the temperature condition of 4°C. The sediments were collected as Niuxin plum wine polysaccharides (NPWP). NPWP solution (250 mL) was separated by the MinimatePall ultrafiltration system (Guangzhou Ewell Bio-Technology Co., Guangzhou, Guangdong, China) with 1,000, 500, 300, 100, 50, 10, 5, 3, and 0.65 kDa-ultrafiltration membranes. Ten kinds of NPWP solutions (50 mL) were mixed with 450 mL of absolute ethanol and preserved under the temperature condition of 4°C for 24 h. All the sediments collected were freeze-dried. The antioxidant and anti-hyperglycemic activities of 10 NPWPs were detected using the methods described below. The NPWP possessing the highest bioactivities were selected to identify the chemical structure.

#### Determination of Monosaccharide Composition

The monosaccharides from NPWP were hydrolyzed and released by the modified method (22). The mixed standard solutions of mannose, rhamnose, glucuronic acid, galacturonic acid, glucose, galactose, and arabinose were prepared by dissolving in distilled water. Monosaccharide composition of NPWP was detected with the Waters e2695 HPLC system (Waters, Milford, MA, USA) coupled with Waters 2695–2998 UV-Detector, and Agilent 5 TC-C18 column (250 mm × 4.6 mm, Agilent Technologies, Palo Alto, CA, USA). The detector wavelength was fixed at 250 nm. Mobile phase A consisted of 15% acetonitrile-0.02 M ammonium acetate, and mobile phase B was 20% acetonitrile-0.02 M ammonium acetate. Flow rate was 5 mL/min. The gradient elution was conducted as follows: 0–25 min, 100% A; 25–40 min, 37.5% A; 40–41 min, 20% A; 41–46 min, 0% A; 46–50 min, 100% A. The temperature of the column was set at 25°C.

#### Determination of FT-IR and NMR Spectra

The NPWP was mixed with KBr, and the mixture was refined by grinding and pressing (23). The scanning analysis was performed with Nicolet Nexus 470 infrared spectrometer (Thermo Electron Co., Waltham, MA, USA) in 4,000–400 cm^−1^. ^1^H NMR (600 MHz) and ^13^C NMR (150 MHz) spectra of NPWP were recorded using Bruker Advance 600M spectrometer (Bruker Co., Rheinstetten, Germany), with deuterated H_2_O (D_2_O) as the solvent and tetramethylsilane (TMS) as the intrinsic criterion. Chemical shifts (δ) were expressed as ppm in contrast to TMS (24).

### Quantification of Minor Bioactive Components (Polyphenols and Alkaloids) in Niuxin Plum Wine

#### Determination of Polyphenol Content

The total phenolic content (TPC) of Niuxin plum wine was assessed following the improved Folin-Ciocalteu assay (25). TPC was expressed as milligrams of gallic acid equivalents (mg GAE/mL wine sample). The total flavonoid content (TFC) of Niuxin plum wine was determined by a modified colorimetric method (26), and the results were expressed as milligrams of rutin equivalents (mg of RE/mL wine sample). The condensed tannin content (CTC) of Niuxin plum wine was measured by a colorimetric method (27). CTC was expressed as milligrams of gallic acid equivalents (mg of GAE/mL wine sample). Monomeric anthocyanin content (MAC) of Niuxin plum wine was measured by a pH differential method with slight modifications (28), and expressed as cyanidin-3-glucoside equivalents (mg Cy3glc/mL wine sample). The total quercetin content of Niuxin plum wine was determined by a modified method (29).

#### Determination of Alkaloid Content

The total alkaloid content (TAC) of Niuxin plum wine was analyzed by the modified method (30). TAC was expressed as milligrams of 4-hydroxypiperidine equivalents (mg of HE/mL Niuxin plum wine sample).

### Analyses on the Functional Activities of Niuxin Plum Wine

#### DPPH Radical-Scavenging Activity

DPPH radical-scavenging activity in Niuxin plum wine was assessed with an improved method (31). The percentage of DPPH discoloration (%) was calculated using the following equation: [1 – (A_sample_ – A_blank_)/A_negative control_] × 100%.

#### Determination of Anti-hyperglycemic Activity

The α-glucosidase-inhibitory activity of Niuxin plum wine was evaluated by using PNPG as the substrate (32). Data concerning enzymatic inhibition has been computed using the following inhibition ratio (%) formula: [1 – (A_2_ – A_3_)/A_1_] × 100%, in which A_1_ is blank absorbance, A_2_ is sample or acarbose absorbance, and A_3_ is the absorbance of sample short of α-glucosidase and PNPG.

### Statistical Analysis

Data present the mean and standard deviation of three replicates. Statistical research was carried out by variance analysis (ANOVA) and SPSS 17.0 statistical software (SPSS Inc., Chicago, IL, USA). Duncan's test was the most effective solution for detecting the notable discrepancy of means (*P* < 0.05).

## Results and Discussion

### Optimal Fermentation Conditions for Niuxin Plum Wine

**Figure 1:**
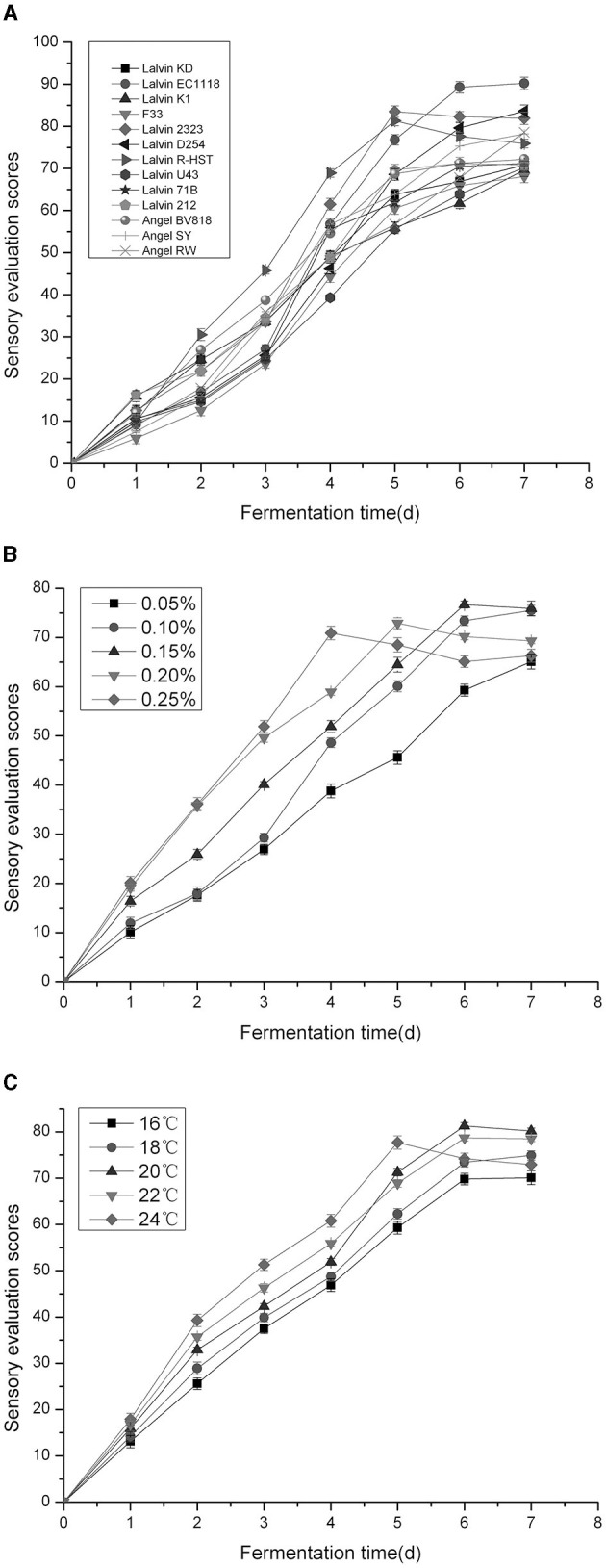
Effects of different *Saccharomyces cerevisiae* strains **(A)**, yeast amounts **(B)**, fermentation temperatures **(C)** on sensory evaluation scores of Niuxin plum wine.

According to Figure 1A, as fermentation progressed, the sensory evaluation scores (SES) of plum wine kept improving until it was balanced. Between days 2 and 4, the SES of plum wines brewed by Lalvin R-HST was highest. On day 5, Lalvin 2323 and Lalvin R-HST plum wines gave the maximum SES, separately. The plum wines brewed by Lalvin EC1118, Lalvin D254, and Lalvin 2323 possessed relatively higher SES at the end of fermentation. These results attribute *Saccharomyces cerevisiae* strain variety as the key factor influencing the quality of the wine. Figure 1B further illustrates that the SES of plum wine brewed by discrepant yeast amounts progressively increased as the duration of fermentation prolonged, and eventually balanced out or declined. Among the five additive concentrations tested, fermentation systems at 0.25% yeast amount approached maximum SES at the earliest (on day 4), followed by 0.20% yeast amount (on day 5) and 0.15% yeast amount (on day 6). On completion of fermentation, the SES of plum wines fermented by 0.10, 0.15, and 0.20% yeast amounts were higher (*P* < 0.05) than others, implying that the yeast amount acts as a significant factor. As seen in Figure 1C, the SES of Niuxin plum wine fermented at different temperatures gradually increased, with the highest SES on day 5 at 24°C, followed by the highest SES of plum wine fermented on day 6 or even day 7 at 20°C. After fermentation was complete, the SES of plum wines fermented at 18, 20, and 22°C were greater (*P* < 0.05) than others, which indicated that temperature plays a crucial role in fermentation.

Based on the findings of single-factor testing, we considered *Saccharomyces cerevisiae* strain (A), yeast amount (B), and fermentation temperature (C) as the significant influence factors fit for orthogonal testing. According to Table 3, Range A > Range B > Range C indicating *Saccharomyces cerevisiae* strain as the foremost influence factor of plum wine SES. The ANOVA of orthogonal experiment results also confirmed that. In comparing the values of three factors, namely k1, k2, and k3, the optimal combination of A1B1C2 was determined. It meant that the optimal conditions of fermentation for plum wine should be *Saccharomyces cerevisiae* strain of Lalvin EC1118, yeast amount of 0.10%, and fermentation temperature of 20°C. Under these conditions, the SES of plum wine was 92.0 ± 0.5, and alcohol content of the produced wine was 13.1% vol.

**Table 3:** Orthogonal experiment results for optimal fermentation conditions of Niuxin plum wines.

**Test No.**	**A**	**B**	**C**	**D**	**Sensory evaluation scores**	**Variance analysis of orthogonal results**					
						**Factor**	**Sum of squares of deviations**	**Free**	**F**	**Critical value of F**	**Significance**
1	1	1	1	1	88.500	A	224.336	2	26.742	19.000	^*^
2	1	2	2	2	91.900	B	127.069	2	15.147	19.000	
3	1	3	3	3	85.000	C	123.529	2	14.725	19.000	
4	2	1	2	3	87.100	Error	8.390	2			
5	2	2	3	1	75.900						
6	2	3	1	2	66.500						
7	3	1	3	2	83.700						
8	3	2	1	3	77.000						
9	3	3	2	1	80.200						
K1	265.400	259.300	232.000	244.600							
K2	229.500	244.800	259.200	242.100							
K3	240.900	231.700	244.600	249.100							
k1	88.467	86.433	77.333	81.533							
k2	76.500	81.600	86.400	80.700							
k3	80.300	77.233	81.533	83.033							
Range	11.967	9.200	9.067	2.333							

*A: Saccharomyces cerevisiae strains (level 1, Lalvin EC1118; level 2, Lalvin D254; level 3, Lalvin 2323), B: Saccharomyces cerevisiae amount (level 1, 0.10%; level 2, 0.15%; level 3, 0.20%), C: Fermentation temperature (level 1, 18°C; level 2, 20°C; level 3, 22°C), and D: Null column*.

* *: Significant difference between F value and Critical value of F*.

### Nutritional Components in Niuxin Plum Wine

The total amino acid content of Niuxin plum wine was detected as 37.99 ± 0.05 mg/100 mL. Among the 17 amino acids found in plum wine, glutamic acid (5.90 ± 0.02 mg/100 mL) was the most abundant (*P* < 0.05), followed by alanine (3.77 ± 0.01 mg/100 mL), aspartic acid (3.74 ± 0.02 mg/100 mL) and proline (2.77 ± 0.02 mg/100 mL). Seven essential amino acids accounted for 33.43% of the total amino acid content, and included threonine (1.57 ± 0.01 mg/100 mL), valine (2.69 ± 0.03 mg/100 mL), methionine (2.45 ± 0.02 mg/100 mL), isoleucine (1.46 ± 0.04 mg/100 mL), leucine (2.42 ± 0.03 mg/100 mL), phenylalanine (0.98 ± 0.05 mg/100 mL) and lysine (1.13 ± 0.01 mg/100 mL). There also existed a small fraction of histidine (0.61 ± 0.03 mg/100 mL), arginine (1.11 ± 0.03 mg/100 mL), glycine (2.32 ± 0.01 mg/100 mL), serine (2.02 ± 0.03 mg/100 mL), tyrosine (1.03 ± 0.02 mg/100 mL) and cysteine (2.02 ± 0.01 mg/100 mL) in Niuxin plum wine.

In Niuxin plum wine, the inductively coupled plasma mass spectrometry with external standard method, was used for the determination of mineral elements. The most widely distributed (*P* < 0.05) element was potassium (123.38 ± 0.01 mg/100 g), followed by calcium (20.54 ± 0.02 mg/100 g), magnesium (8.30 ± 0.01 mg/100 g), zinc (4.60 ± 0.03 mg/100 g), sodium (1.63 ± 0.03 mg/100 g), manganese (0.29 ± 0.02 mg/100 g), boron (0.06 ± 0.01 mg/100 g), barium (0.05 ± 0.01 mg/100 g) and strontium (0.03 ± 0.01 mg/100 g).

In Niuxin plum wine, four important vitamins were determined selectively through qualitative and quantitative analysis. Among them, vitamin C (590 ± 0.01 μg/100 mL) exhibited the maximum content (*P* < 0.05), and it was successively followed by vitamin E (6.96 ± 0.01 μg/100 mL), vitamin B9 (0.33 ± 0.01 μg/100 mL) and vitamin A (<0.1 μg/100 mL).

### Volatile Aromas in Niuxin Plum Wine

In fruit wine, it is a well-known fact that compounds with volatile properties affect the flavor perception and sensory quality (33). As seen in Table 4, a total of 55 volatile compounds were identified in Niuxin plum wine by GC, including 28 esters, 11 alcohols, 4 alkenes, 3 aldehydes, 3 acids, 2 alkanes, 2 phenols, 1 ketone, and 1 naphthalene. The major volatile constituent with retention time of 4.706 min was clearly identified as 2-hydroxy-propanoic acid ethyl ester (70.511%). Other volatile substances with retention time (min) of 16.605, 11.544, 8.701, and 19.608 corresponded, respectively, to ethyl octanoate (15.645%), isoamyl alcohol (3.372%), 3-methyl butyl acetate (3.333%), and ethyl decanoate (2.908%).

**Table 4:** Volatile aroma contents in Niuxin plum wine.

**Retention time (min)**	**Peak name**	**CAS Number**	**Area (%)**	**F. Match**	**R. Match**
3.573	Acetic acid ethyl ester	141-78-6	0.624	919	919
4.706	2-Hydroxy-propanoic acid ethyl ester	97-64-3	70.511	888	888
6.006	2-Methylpropyl acetate	110-19-0	0.025	763	800
6.582	Butanoic acid ethyl ester	105-54-4	0.022	926	946
8.488	2-Methyl-1-propanol	78-83-1	0.762	889	932
8.701	3-Methylbutyl acetate	123-92-2	3.333	969	969
10.062	Decamethylcyclopentasiloxane	541-02-6	0.004	956	960
11.544	Isoamyl alcohol	123-51-3	3.372	928	928
11.883	Ethyl hexanoate	123-66-0	0.935	961	961
12.454	Bicyclo (4.2.0) octa-1,3,5-triene	694-87-1	0.012	919	951
12.995	Hexyl acetate	142-92-7	0.147	945	945
13.941	(Z)-3-Hexen-1-ol acetate	3681-71-8	0.007	948	948
14.147	4-Hexen-1-ol acetate	72237-36-6	0.097	959	959
14.930	Isobutyl hexanoate	105-79-3	0.002	794	869
15.092	1-Hexanol	111-27-3	0.074	931	931
15.294	(E)-3-Hexen-1-ol	928-97-2	0.005	875	889
15.693	(Z)-4-Octenoicacid methyl ester	21063-71-8	0.093	813	838
16.345	2-Octanol	123-96-6	0.022	914	914
16.605	Ethyl octanoate	106-32-1	15.645	931	936
16.824	Acetic acid	64-19-7	0.059	962	962
16.950	Isopentyl hexanoate	2198-61-0	0.024	915	915
17.032	2-Furancarboxaldehyde	98-01-1	0.009	912	940
17.448	Isopropyl pentakis (trimethylsilyl) diorthosilicate	71579-69-6	0.003	773	773
17.495	Methyl non-anoate	1731-84-6	0.007	810	814
17.589	Decanal	112-31-2	0.004	952	952
17.915	Propyl octanoate	624-13-5	0.012	942	952
17.998	Benzaldehyde	100-52-7	0.082	973	973
18.162	Ethyl non-anoate	123-29-5	0.048	955	960
18.327	Linalool	78-70-6	0.007	888	888
18.396	Butylcaprylate	589-75-3	0.018	936	936
18.531	1-Octanol	111-87-5	0.006	886	912
18.681	3-Methyl-2-hexanol	2313-65-7	0.011	752	845
19.008	Decanoic acid methyl ester	110-42-9	0.013	946	946
19.533	γ-Butyrolactone	96-48-0	0.008	759	834
19.608	Ethyl decanoate	110-38-3	2.908	965	970
19.855	3-Methylbutyl octanoate	2035-99-6	0.065	940	940
19.907	1-Nonanol	143-08-8	0.033	947	953
20.026	Ethyl benzoate	93-89-0	0.07	938	942
20.080	Diethyl butanedioate	123-25-1	0.01	920	947
20.133	4-Methoxystyrene	637-69-4	0.029	938	938
20.396	(-)-β-Fenchyl alcohol	470-08-6	0.004	904	907
21.032	1,1,6-Trimethyl-1,2-dihydronaphthalene	30364-38-6	0.009	899	913
21.393	Methyl salicylate	119-36-8	0.010	897	931
21.457	Ethyl phenylacetate	101-97-3	0.004	801	801
21.816	2-Phenylethyl acetate	103-45-7	0.078	956	956
21.911	β-Damascenone	23726-93-4	0.003	914	914
22.055	Ethyl dodecanoate	106-33-2	0.026	966	970
22.096	Hexanoic acid	142-62-1	0.040	958	958
22.542	Benzyl alcohol	100-51-6	0.016	954	954
22.971	Phenylethyl Alcohol	60-12-8	0.343	970	970
24.338	Octanoic acid	124-07-2	0.157	960	960
25.129	Ethyl cinnamate	103-36-6	0.004	920	925
25.428	Eugenol	97-53-0	0.005	927	928
26.155	Ethyl hexadecanoate	628-97-7	0.006	923	923
26.634	2,4-Bis (1,1-dimethylethyl)-phenol	96-76-4	0.016	944	944

### Identification of Major Functional Components in Niuxin Plum Wine

#### Determination of Major Functional Components

The intake of fruit wines such as grape wine, in moderation, can decrease the risk of coronary heart diseases (34). In general, the functional components of grape wine include phenols (1.207–4.263 mg GAE/mL), flavonoids (0.007–3.489 mg RE/mL), tannins (0.636–2.355 mg/mL) (35), anthocyanins (0–0.827 mg/mL), quercetin (0–0.0077 mg/mL) (36), and polysaccharides (0.282–1.600 mg/mL) (37). In this study, Niuxin plum wine possessed phenols (1.601 ± 0.001 mg GAE/mL), flavonoids (2.453 ± 0.003 mg RE/mL), tannins (1.036 ± 0.002 mg GAE/mL), anthocyanins (1.070 ± 0.010 mg Cy3glc/mL), quercetin (0.001 ± 0.0002 mg/mL), and polysaccharides (0.729 ± 0.010 mg GE/mL) as functional components. The producted wine also possessed alkaloid value with 0.282 ± 0.001 mg HE/mL. As compared to grape wine, Niuxin plum wine possessed 1.294 times higher anthocyanin content than grape wine. Whereas, phenolic, flavonoid, tannin, anthocyanin and quercetin contents, as well as polysaccharide content in plum wine fell in the range of those in grape wine.

The antioxidant and hypoglycemic activities of polyphenols, alkaloids, and polysaccharides in Niuxin plum wine were verified preliminarily. Phenolic extracts from Niuxin plum wine scavenged activity against DPPH radicals with 40.362 ± 1.171% and inhibited α-glucosidase activity with 7.726 ± 0.842%, as well as alkaloid extracts from Niuxin plum wine possessed DPPH radical scavenging activity (43.692 ± 1.915%) and inhibiting α-glucosidase capacity (6.918 ± 0.350%). However, polysaccharides extracted from Niuxin plum wine exhibited significantly higher DPPH radical scavenging activity (77.646 ± 0.916%) and α-glucosidase inhibitory capacity (50.446 ± 0.826%) than those of both phenolic and alkaloid extracts (*P* < 0.05). Therefore, polysaccharides are considered as the major functional components in Niuxin plum wine, and were further isolated for selecting the fraction with the highest antioxidant and hypoglycemic activities, illustrating its structural characterization and analyzing the structure-activity relationship.

#### Selection of NPWPs With the Highest Bioactivities

**Figure 2:**
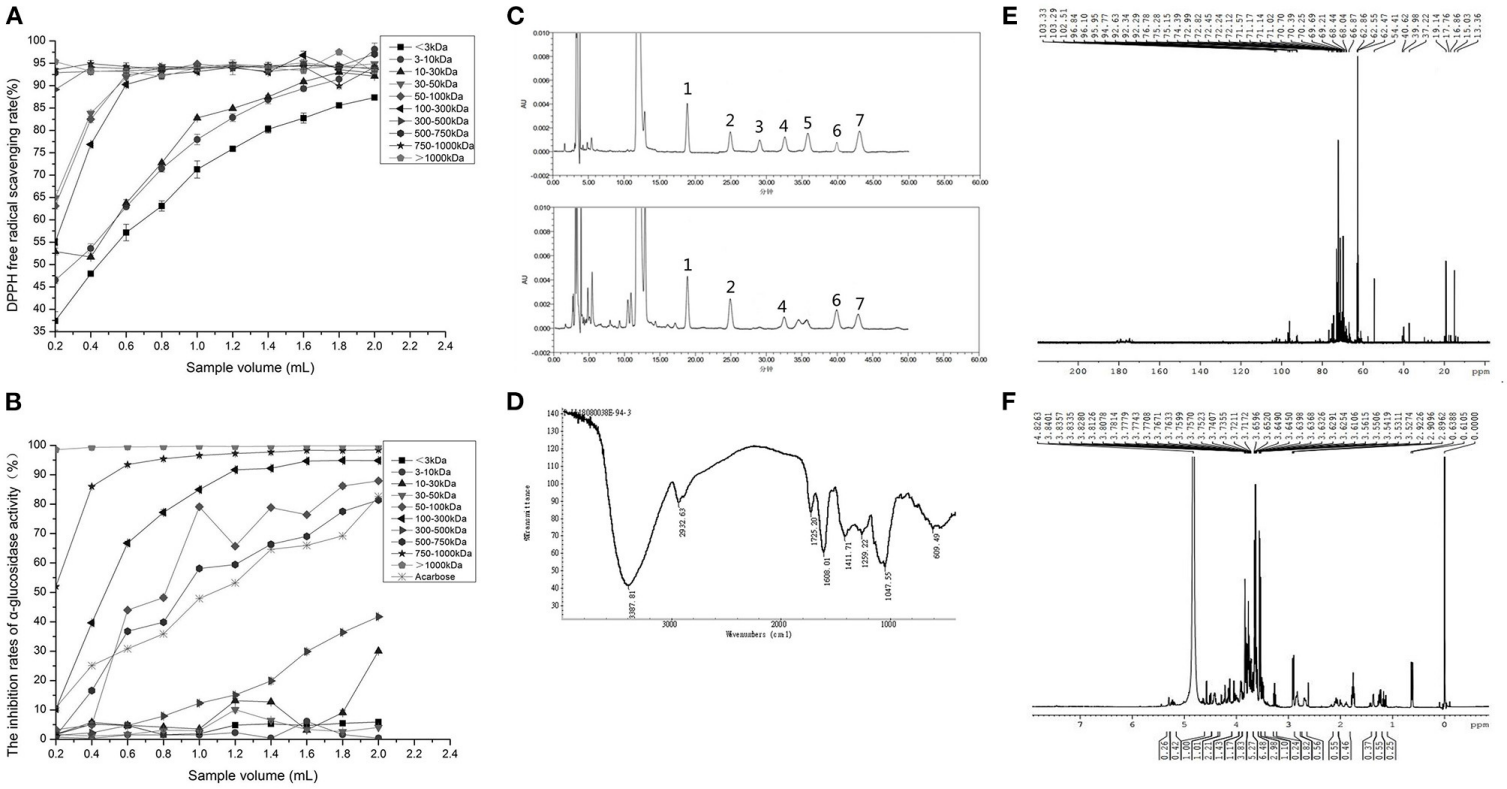
Functional activities and chemical structures of Niuxin plum wine polysaccharides (NPWPs). DPPH radical-scavenging rates of NPWPs with different molecular weight **(A)**, anti-hyperglycemic activities of acarbose and NPWPs with different molecular weights **(B)**, HPLC chromatogram of mixed monosaccharide standards and monosaccharide compositions of NPWPs **(C)**, (1: mannose, 2: rhamnose, 3: glucuronic acid, 4: galacturonic acid, 5: glucose, 6: galactose, and 7: arabinose), infrared spectrum of NPWP with molecular weight >1,000 kDa **(D)**, ^13^C NMR spectrum of NPWP with molecular weight >1,000 kDa **(E)**, and ^1^H NMR spectrum of NPWP with molecular weight >1,000 kDa **(F)**.

After isolation and purification, 10 NPWPs were obtained, having molecular weights of <3 kDa (NPWP-1), 3–10 kDa (NPWP-2), 10–30 kDa (NPWP-3), 30–50 kDa (NPWP-4), 50–100 kDa (NPWP-5), 100–300 kDa (NPWP-6), 300–500 kDa (NPWP-7), 500–750 kDa (NPWP-8), 750–1,000 kDa (NPWP-9), and >1,000 kDa (NPWP-10). According to Figure 2A, 10 NPWPs had DPPH radical scavenging capacities. With the increase in sample volume, the scavenging capacities of 10 NPWPs slowly improved and eventually tapered to form a flat line. It is worth noting that NPWP-7, NPWP-8, NPWP-9, and NPWP-10 maintained high scavenging activities, and NPWP-10 showed relatively higher DPPH radical scavenging capacity (from 92.06 to 97.51%) than others.

In the present study, the anti-hyperglycemic abilities of 10 NPWPs were detected using acarbose as control. From Figure 2B, it can be seen that the 10 NPWPs had significantly different α-glucosidase inhibitory capabilities. With the rise in sample volume, NPWP-1, NPWP-2, NPWP-3, NPWP-4, and NPWP-7 exhibited a slight increase in the inhibition activities; however, the extent of increase was far lower than the control. In contrast, NPWP-5, NPWP-6, NPWP-8, NPWP-9, and NPWP-10 showed a rapid increase in the inhibition activities and eventually were higher than control. NPWP-10 had the highest α-glucosidase inhibitory capacity (above 95%) among all NPWPs.

#### Structural Identification of NPWP With the Highest Bioactivities

The HPLC chromatograms of monosaccharides hydrolyzed from NPWP-10 along with the seven standards are shown in Figure 2C. NPWP-10 was mainly composed of mannose, rhamnose, arabinose, galactose, and galacturonic acid at a molar ratio of 2.570: 1.775: 1.045: 1.037: 1, indicating that NPWP-10 is a heteropolysaccharide with mannose as the predominant monosaccharide component.

The FT-IR spectrum of NPWP-10 was shown in Figure 2D. The peak of absorption occurred at 3,387.81 cm^−1^, possibly as a result of the O–H bond's stretching vibration on carboxylic acids. The absorption peak at 2,932.63 cm^−1^ should be attributable to the C–H bond's stretching vibration. Absorption peaks at 1,725.20 cm^−1^ and 1,608.01 cm^−1^ were associated with symmetrical and asymmetrical stretching vibration of the carboxyl (–COOH) C = O bond. The absorption peak at 1,411.71 cm^−1^ might be caused by the stretching vibration of C–N in the amide bond. The stretching vibration of the C–O bond's caused the absorption peak at 1,259.22 cm^−1^. A strong absorption peak was observed at 1,047.55 cm^−1^, indicating that this NPWP contained a pyranose ring and had the stretching vibration of the C–O–C bond. The pyranose ring's symmetrical stretching vibration caused the absorption peak at 609.49 cm^−1^.

According to the ^13^C NMR spectrum of NPWP-10 (Figure 2E), several signals were located between δ 90 and δ 102, suggesting that sugar-ring was mainly α-configuration. A small number of signals appeared in the area of δ 102–112, illustrating a β-conformation in this NPWP. Signals across the area of δ 82–84 indicated the presence of pyranose structure. Only one signal appeared in the region from δ 76 to δ 85, and thirteen signals appeared across the area in δ 70–75, indicating that there was almost no substitution of C2, C3, and C4 carbons of pyranose. Three distinct signals appeared at δ 62.47, δ 62.55, and δ 62.86 but not near δ 67, supporting the fact that pyranose C6 was not replaced. Because of the presence of methyl in 6-deoxy sugar, two signals appeared in δ 16.86 and δ 17.76, respectively. As ^1^H NMR spectra of NPWP-10 (Figure 2F), the signal δ 4.82 fell in the anomeric proton region (δ 4.3–5.9), which was situated at a higher field (δ < 5), indicating that the sugar-ring was β-configuration.

#### Structure-Activity Relationship of NPWP

The chemical structure of active polysaccharide is the basis of its biological activity such as antioxidant and antidiabetic activities. Molecular weight, monosaccharide composition, branching degrees and functional groups, as well as glycosidic linkages play important roles on the bioactivities of polysaccharides (38). Generally, polysaccharides of molecular weight over 90 kDa usually own the formation of advanced confirmation and triple helix structure, which are important for high bioactivities (39). In this study, with the increase in molecular weight, the NPWPs' bioactivities improved continuously, and NPWP-10 exhibited the highest DPPH radical scavenging activity and α-glucosidase inhibitory capability.

Monosaccharide composition is also partially responsible for variations in bioactivities of polysaccharides. Polysaccharides containing mannose and rhamnose exhibit more potent bioactivities than polysaccharides without those compositions (40). Through the analysis of monosaccharide compositions, NPWP-10 was primarily made up of mannose, rhamnose, arabinose, galactose and galacturonic acid. Among them, mannose content has the highest value, followed by rhamnose content, which could be considered as one reason for antioxidant and hypoglycemic activities of NPWP-10. In addition, the solubility of polysaccharides in water influences their bioactivities as well. The water solubility varied based on the uronic acid content of polysaccharides. High uronic acid content indicated superior water solubility of polysaccharides (41). Through the analysis of monosaccharide compositions, galacturonic acid was one of the main components in NPWP-10, which enhanced the water solubility and improved the bioactivities.

Moreover, the configuration of sugar chains is specifically associated with bioactivities of polysaccharides. The structure of sugar-ring with β-configuration could effectively avoid the degradation of α-glucosidase in the human body and exert its biological activities (42). FT-IR and NMR spectra confirmed that NPWP-10 contained α-configuration as the main component and β-configuration as the auxiliary component. The existence of β-configuration played a specific role in enhancing bioactivities of NPWP-10.

## Conclusions

The optimal fermentation conditions for Niuxin plum wine include *Saccharomyces cerevisiae* strain of EC1118, yeast amount of 0.1%, and fermentation temperature of 20 °C. We detected 17 amino acids, 9 mineral elements, 4 vitamins, and 55 volatile compounds in wine, with the significant functional components being polysaccharides. NPWP-10 had the highest antioxidant and anti-hyperglycemic activities among all NPWPs. It was mainly composed of mannose, rhamnose, arabinose, galactose, and galacturonic acid, in a molar ratio of 2.570: 1.775: 1.045: 1.037: 1. NPWP-10 contained α-configuration as the main component and β-configuration as the auxiliary component. Functional activities *in vivo* and *in vitro* of NPWP-10 from Niuxin plum wine needs to be investigated further. Moreover, the special fermentation technique of Niuxin plum wine from this research needs to be improved for industrial application further, based on the characteristics of the fruit variety.

## Data Availability Statement

The original contributions presented in the study are included in the article/supplementary material, further inquiries can be directed to the corresponding author/s.

## Author Contributions

GL, PW, YT, CR, CW, XH, LL, and XC conducted experimental design and carried out the experiment. GL, YT, and JS prepared the manuscript. YP edited the revised manuscript. JL and DL contributed helpful discussion and scientific advice during the preparation of manuscript. All authors contributed to the article and approved the submitted version.

## Funding

The program was funded by Special Fund for Guangxi Bagui Scholars [Grant No. (2016) 21], Guangxi Scientific Research and Technological Development Projects (Grant No. Gui Ke AD19110141), and Foundation of Fundamental Research Project from Guangxi Academy of Agricultural Sciences (Grant Nos. 2021YT111, 2021YT112, 2021YT113, and 2021YTI16).

## Conflict of Interest

The authors declare that the research was conducted in the absence of any commercial or financial relationships that could be construed as a potential conflict of interest.

## Publisher's Note

All claims expressed in this article are solely those of the authors and do not necessarily represent those of their affiliated organizations, or those of the publisher, the editors and the reviewers. Any product that may be evaluated in this article, or claim that may be made by its manufacturer, is not guaranteed or endorsed by the publisher.

## References

[1] An K Wu J Tang D Wen J Fu M Xiao G. Effect of carbonic maceration (CM) on mass transfer characteristics and quality attributes of Sanhua plum (*Prunus salicina* Lindl.). Int J Food Sci Technol. (2018) 87:537–45. 10.1016/j.lwt.2017.09.032

[2] Lozano M Vidal-Aragón MC Hernández MT Ayuso MC Bernalte MJ García J. Physicochemical and nutritional properties and volatile constituents of six Japanese plum (*Prunus salicina* Lindl.) cultivars. Eur Food Res Technol. (2009) 228:403–10. 10.1007/s00217-008-0946-3

[3] Louw ED Theron KI. Volatile dynamics during maturation, ripening and cold storage of three Japanese plum cultivars (*Prunus salicina* Lindl.). Postharvest Biol Tec. (2012) 70:13–24. 10.1016/j.postharvbio.2012.03.007

[4] Cuevas FJ Moreno-Rojas JM Arroyo F Daza A Ruiz-Moreno MJ. Effect of management (organic vs. conventional) on volatile profiles of six plum cultivars (Prunus salicina Lindl.). A chemometric approach for varietal classification and determination of potential markers. Food Chem. (2016) 199:479–84. 10.1016/j.foodchem.2015.12.049 26775998

[5] de Beer D Steyn N Joubert E Muller N. Enhancing the polyphenol content of a red-fleshed Japanese plum (*Prunus salicina* Lindl.) nectar by incorporating a polyphenol-rich extract from the skins. J Sci Food Agric. (2012) 92:2741–50. 10.1002/jsfa.5704 22522565

[6] Roca M Ríos JJ Chahuaris A Pérez-Gálvez A. Non-fluorescent and yellow chlorophyll catabolites in Japanese plum fruits (*Prunus salicina* Lindl.). Food Res Int. (2017) 100:332–8. 10.1016/j.foodres.2017.07.029 28964356

[7] Venter A Joubert E de Beer D. Characterisation of phenolic compounds in South African plum fruits (*Prunus salicina* Lindl.) using HPLC coupled with diode-array, fluorescence, mass spectrometry and on-line antioxidant detection. Molecules. (2013) 18:5072–90. 10.3390/molecules18055072 23644975 PMC6270170

[8] Joshi SS Kuszynski CA Bagchi M Bagchi D. Chemopreventive effects of grape seed proanthocyanidin extract on Chang liver cells. Toxicology. (2000) 155:83–90. 10.1016/S0300-483X(00)00280-8 11154800

[9] Li YM Zhong RF Chen J Luo ZG. Structural characterization, anticancer, hypoglycemia and immune activities of polysaccharides from *Russula virescens*. Int J Biol Macromol. (2021) 184:380–92. 10.1016/j.ijbiomac.2021.06.026 34126149

[10] Sun YQ Huo JX Zhong S Zhu JX Li YG Li XJ. Chemical structure and anti-inflammatory activity of a branched polysaccharide isolated from *Phellinus baumii*. Carbohyd Polym. (2021) 268:118214. 10.1016/j.carbpol.2021.118214 34127216

[11] Miljić U Puškaš V Vučurović V. Investigation of technological approaches for reduction of methanol formation in plum wines. J Inst Brew. (2016) 122:635–43. 10.1002/jib.376

[12] Gülcü M Uslu N Özcan MM Gökmen F Özcan MM Banjanin T. The investigation of bioactive compounds of wine, grape juice and boiled grape juice wastes. J Food Process Pres. (2019) 43:e13850. 10.1111/jfpp.13850

[13] Xu JN Qi YM Zhang J Liu MM Wei XY Fan MT. Effect of reduced glutathione on the quality characteristics of apple wine during alcoholic fermentation. Food Chem. (2019) 300:125130. 10.1016/j.foodchem.2019.125130 31325746

[14] Cakara U Petrovićb A Pejinc B Cakara M Živkovićd M Vajsd V. Fruit as a substrate for a wine: a case study of selected berry and drupe fruit wines. Sci Hortic. (2019) 244:42–49. 10.1016/j.scienta.2018.09.020

[15] Zhen D Lv M Chen MB Luo JJ Liu DQ. Effects of a mutated yeast plus addition of sucrose and nitrogen on the total higher alcohol levels of a plum wine fermentation. J Inst Brew. (2014) 120:571–4. 10.1002/jib.163

[16] Jaiswal R Karakose H Ruhmann S Goldner K Neumuller M Treutter D. Identification of phenolic compounds in plum fruits (*Prunus salicina* L. and Prunus domestica L.) by high-performance liquid chromatography/tandem mass spectrometry and characterization of varieties by quantitative phenolic fingerprints. J Agric Food Chem. (2013) 61:12020–31. 10.1021/jf402288j 24152059

[17] Galgano F Favati F Caruso M Scarpa T Palma A. Analysis of trace elements in southern Italian wines and their classification according to provenance. Int J Food Sci Technol. (2008) 41:1808–15. 10.1016/j.lwt.2008.01.015

[18] Pozo-Bay?n MA G-Alegría E Polo MC Tenorio C Martín-Álvarez PJ Calvo de la Banda MT. Wine volatile and amino acid composition after malolactic fermentation: effect of *Oenococcus oeni* and *Lactobacillus plantarum* starter cultures. J Agr Food Chem. (2005) 53:8729–35. 10.1021/jf050739y 16248578

[19] Santos J Mendiola JA Oliveira MBPP Ibánez E Herrero M Sequential determination of fat- water-soluble vitamins in green leafy vegetables during storage. J Chromatogr A. (2012) 1261:179–88. 10.1016/j.chroma.2012.04.067 22608116

[20] Tang ZS Zeng XA Brennan MA Han Z Niu DB Huo YJ. Characterization of aroma profile and characteristic aromas during lychee wine fermentation. J Food Process Pres. (2019) 43:e14003. 10.1111/jfpp.14003

[21] Zhang H Zou P Zhao HT Qiu JQ Regenstein JM Yang X. Isolation, purification, structure and antioxidant activity of polysaccharide from pinecones of *Pinus koraiensis*. Carbohyd Polym. (2021) 251:117078. 10.1016/j.carbpol.2020.117078 33142621

[22] Liu GM Sun J He XM Tang YY Li JM Ling DN. Fermentation process optimization and chemical constituent analysis on longan (*Dimocarpus longan* Lour.) wine. Food Chem. (2018) 256:268–79. 10.1016/j.foodchem.2018.02.064 29606448

[23] Hu GT Zou XY Li EN Liao TS Wu H Wen P. Effects of enzymatic hydrolysis on the structural, rheological, and functional properties of mulberry leaf polysaccharide. Food Chem. (2021) 355:129608. 10.1016/j.foodchem.2021.129608 33799260

[24] Chen C Wang PP Huang Q You LJ Liu RH Zhao MM. A comparison study on polysaccharides extracted from *Fructus Mori* using different methods: structural characterization and glucose entrapment. Food Funct. (2019) 10:3684–95. 10.1039/C9FO00026G 31168531

[25] Kumar S Narwal S Kumar V Prakash O. α-glucosidase inhibitors from plants: A natural approach to treat diabetes. Pharmacogn Rev. (2011) 5:19–29. 10.4103/0973-7847.79096 22096315 PMC3210010

[26] Borkataky M Kakoty B Saikia L. Influence of total phenolic content and total flavonoid content of the DPPH radical scavenging activity of *Eclipta Alba* (L.) Hassk. Int J Pharm Pharm Sci. (2013) 5:224–327.

[27] Xu BJ Chang SKC. A comparative study on phenolic profiles and antioxidant activities of legumes as affected by extraction solvents. J Food Sci. (2007) 72:S159–66. 10.1111/j.1750-3841.2006.00260.x 17995858

[28] Tang YY Cai WX Xu BJ. From rice bag to table: fate of phenolic chemical compositions and antioxidant activities in waxy and non-waxy black rice during home cooking. Food Chem. (2016) 191:81–90. 10.1016/j.foodchem.2015.02.001 26258705

[29] Eftekhari M Alizadeh M Ebrahimi P. Evaluation of the total phenolics and quercetin content of foliage in mycorrhizal grape (*Vitis vinifera* L.) varieties and effect of postharvest drying on quercetin yield. Ind Crop Prod. (2012) 38:160–5. 10.1016/j.indcrop.2012.01.022

[30] Ganga Rao B Umamaheswara Rao P Sambasiva Rao E Mallikarjuna Rao T Praneeth D. Studies on phytochemical constituents, quantification of total phenolic, alkaloid content and *in-vitro* anti-oxidant activity of *Thespesia populnea* seeds. Free Radic Antioxid. (2011) 1:56–61. 10.5530/ax.2011.4.9

[31] Tang YY He XM Sun J Liu GM Li CB Li L. Comprehensive evaluation on tailor-made deep eutectic solvents (DESs) in extracting tea saponins from seed pomace of *Camellia oleifera* Abel. Food Chem. (2021) 342:128243. 10.1016/j.foodchem.2020.128243 33069529

[32] Tang YY He XM Sun J Li CB Li L Sheng JF. Polyphenols and alkaloids in by-products of longan fruits (*Dimocarpus longan* Lour.) and their bioactivities. Molecules. (2019) 24:1186–01. 10.3390/molecules24061186 30917573 PMC6471414

[33] Mandha J Shumoy H Devaere J Schouteten JJ Gellynck X Winne A. Effect of lactic acid fermentation of watermelon juice on its sensory acceptability and volatile compounds. Food Chem. (2021) 358:129809. 10.1016/j.foodchem.2021.129809 33933966

[34] Renaud S de Lorgeril M. Wine, alcohol, platelets, and the French paradox for coronary heart disease. Lancet. (1992) 339:1523–6. 10.1016/0140-6736(92)91277-F 1351198

[35] Hosu A Cristea VM Cimpoiu C. Analysis of total phenolic, flavonoids, anthocyanins and tannins content in Romanian red wines: prediction of antioxidant activities and classification of wines using artificial neural networks. Food Chem. (2014) 150:113–8. 10.1016/j.foodchem.2013.10.153 24360427

[36] Treptow TC Comarella CG Franco FW Rodrigues E Domingues F Bochi VC. Thermal pest control in ‘Tannat’ grapes: effect on anthocyanins, sensory and color of one-year-old wines. Food Res Int. (2017) 100:113–21. 10.1016/j.foodres.2017.08.011 28888431

[37] Jégou S Hoang DA Salmon T Williams P Oluwa S Vrigneau C. Effect of grape juice press fractioning on polysaccharide and oligosaccharide compositions of *Pinot meunier* and chardonnay champagne base wines. Food Chem. (2017) 232:49–59. 10.1016/j.foodchem.2017.03.032 28490102

[38] Wang JQ Hu SZ Nie SP Yu Q Xie MY. Reviews on mechanisms of *in vitro* antioxidant activity of polysaccharides. Oxid Med Cell Longev. (2016) 5692852:1–3. 10.1155/2016/5692852 26682009 PMC4670676

[39] El Enshasy HA Hatti-Kaul R. Mushroom immunomodulators: unique molecules with unlimited applications. Trends Biotechnol. (2013) 31:668–77. 10.1016/j.tibtech.2013.09.003 24125745

[40] Lo TCT Chang CA Chiu KH Tsay PK Jen JF. Correlation evaluation of antioxidant properties on the monosaccharide components and glycosyl linkages of polysaccharide with different measuring methods. Carbohyd. Polym. (2011) 86:320–7. 10.1016/j.carbpol.2011.04.056

[41] Wu SH Li F Jia SY Ren HT Gong GL Wang YY. Drying effects on the antioxidant properties of polysaccharides obtained from *Agaricus blazei* Murrill. Carbohyd Polym. (2014) 103:414–7. 10.1016/j.carbpol.2013.11.075 24528748

[42] Bluhm TL Sarko A. The triple helical structure of lentinan, a linear β-(l → 3)-D-glucan. Can J Chem. (1977) 55:293–9. 10.1139/v77-044

